# Transactions of the American Laryngological Association

**Published:** 1891-07

**Authors:** 


Transactions of the American Laryngological Association. Twelfth Annual Meeting, Baltimore, May 29-31, 1890. New York : D. Appleton & Co. 1891.

The annual publications of these Transactions of the Association always places in a permanent form much valuable material contributed to the science of Laryngology. The present volume is no exception to the rule. We notice in it many reports of rare forms of disease, besides the usual number of general papers. The officers for 1891 are as follows: President, Dr. W. C. Glasgow, St. Louis; First Vice-President, Dr. John O. Roe, Rochester; Second Vice-President, Dr. J. H. Hartman, Baltimore; Secretary and Treasurer, Dr. Charles H. Knight, New York; Librarian, Dr. T. R. French, Brooklyn. The place of meeting will be in Washington, in connection with the Congress of Physicians and Surgeons in September. F. H. P.

